# SGLT2 inhibitors in hemodialysis or peritoneal dialysis patients: rationale and state-of-the art

**DOI:** 10.1093/ckj/sfaf350

**Published:** 2025-11-14

**Authors:** Roberto Minutolo, Chiara Ruotolo, Giuseppe Conte, Silvio Borrelli

**Affiliations:** Nephrology Unit at University of Campania “Luigi Vanvitelli”, Naples, Italy; Nephrology Unit at University of Campania “Luigi Vanvitelli”, Naples, Italy; Nephrology Unit at University of Campania “Luigi Vanvitelli”, Naples, Italy; Nephrology Unit at University of Campania “Luigi Vanvitelli”, Naples, Italy

**Keywords:** cardiovascular, hemodialysis, peritoneal dialysis, peritoneal membrane, SGLT2 inhibitors

## Abstract

Sodium-glucose cotransporter-2 inhibitors (SGLT2i) reduce the risk of end-stage kidney disease (ESKD), cardiovascular events, and all-cause mortality in chronic kidney disease (CKD), regardless of diabetes or baseline renal function. However, patients with severely impaired kidney function or on dialysis have been excluded from landmark randomized clinical trials (RCTs). Several off-target mechanisms of SGLT2i could be involved in the cardiac protection of patients with ESKD in which the reduced nephron mass strongly diminishes the tubular effects of SGLT2i. However, available evidence in patients undergoing hemodialysis (HD) and peritoneal dialysis (PD) is limited thus leaving efficacy and safety in this population uncertain.

Pharmacokinetic studies confirm that dapagliflozin is not dialyzable and shows no significant accumulation in dialysis patients. Small exploratory trials reported favorable cardiovascular and electrophysiological effects of SGLT2i in HD, while retrospective studies suggest they may preserve residual kidney function in incremental dialysis and improve volume status without major safety concerns. Large retrospective cohort analyses of patients starting dialysis showed lower risks of cardiovascular events and mortality in SGLT2i users compared with non-users. When treated with PD, no study has reported outcome data, and findings on efficacy were mixed with some studies showing increased ultrafiltration and lower blood pressure, while others showed no effect on peritoneal glucose transport.

The theoretic protection against cardiovascular and mortality risk of SGLT2i must be confirmed by ongoing large-scale trials that will clarify the role of this class of drugs in dialysis populations.

## INTRODUCTION

Large placebo-controlled randomized clinical trials (RCTs) have clearly demonstrated the efficacy of sodium‐glucose cotransporter‐2 inhibitors (SGLT2i) in reducing the risk of end-stage kidney disease (ESKD), cardiovascular events and all-cause mortality in people with CKD [1–3], independently from diabetes, primary renal disease, albuminuria level, and eGFR category [4]. Furthermore, SGLT2i are now considered the gold standard of treatment even in populations at high cardiovascular risk without CKD [5, 6]. However, patients with severely impaired kidney function (eGFR <20 ml/min/1.73 m^2^ and those receiving dialysis) have been consistently excluded from the landmark RCTs. This seems reasonable based on the mechanism of action of these drugs. Indeed, SGLT2i block glucose and sodium reabsorption in the proximal tubule, enhancing glycosuria and natriuresis with consequent tubule-glomerular feedback re-activation that in turn reduces intra-glomerular pressure through afferent arteriole vasoconstriction [7, 8]. In patients with low nephron mass, SGLT2i may plausibly be less effective due to the reduced number of tubular SGLT2 transporters.

## RATIONALE OF USE IN END-STAGE KIDNEY DISEASE

The beneficial renal effects of SGLT2i are not exclusively dependent on the hemodynamic mechanism (reduced glomerular hyperfiltration) but they can be mediated by other non-hemodynamic factors involving intra-renal inflammation, renal fibrosis, and oxidative stress [9–11]. The natriuretic effects of SGLT2i, responsible for improvement in blood pressure (BP) and volume overload, can also positively affect cardiovascular protection. Other pleiotropic mechanisms besides tubular action may contribute to cardiac protection [12]. Indeed, experimental studies using a co-culture of human cardiac endothelial cells and cardiomyocytes disclosed that empagliflozin restored the impaired cardiomyocyte relaxation and contraction observed by adding serum from patients on dialysis to cell culture [13]. This beneficial effect seems mediated by a reduction of mitochondrial oxidative damage and enhanced endothelial nitric oxide (NO) bioavailability [13, 14]. Additional SGLT2i protective effects involve regulation of cytosolic calcium and sodium level via inhibition of the sodium-hydrogen exchanger-1 and sodium-calcium exchanger in endothelial cells [15]. Overload of intracellular calcium in endothelial cardiac cells, in fact, stimulates negative signaling pathways, leading to impaired endothelium-derived vasodilation (through a reduction of NO bioavailability), enhanced inflammation, and platelet aggregation. These endothelial protective effects of SGLT2i could be independent of the SGLT2 transporter, indicating that SGLT2i directly target endothelial ion channels such as the sodium-hydrogen exchanger-1 and sodium-calcium exchanger [15]. However, hyperglycemia and micro-inflammation have been shown to increase SGLT2 expression in both human and porcine endothelial cells, therefore supporting the protective role for SGLT2i on the endothelial dysfunction in pathological states [16, 17].

Besides endothelial dysfunction, SGLT2i promotes cardiac benefits through direct cardiomyocyte protection. Indeed, experimental studies evidenced that SGLT2i reduced the infarct size in different animal models with and without diabetes [18, 19], an effect also observed in Sglt2 knockout mice, suggesting independence from SGLT2 expression [20]. More recently, in a mouse model of acute myocardial infarction, it was reported that either pre-treatment or post-reperfusion administration of empagliflozin induced a reduction in infarct size by reverting transcriptomic dysregulation in endothelial cells, fibroblasts, and cardiomyocytes, thus preserving left ventricular function and attenuating inflammatory infiltration [21].

An additional non-hemodynamic mechanism supporting the cardiac protection involves interaction of these drugs with gut microbiome [22]. In a comprehensive omics analysis of a series of experimental studies, Billing *et al*. elegantly demonstrated that dapagliflozin reduced microbiome formation of uremic toxins such as p-cresol sulfate in rats, Sglt2 knockout mice, as well as in patients with diabetes or decompensated heart failure [23]. The improvement of this metabolic picture translates into less proximal tubule glucotoxicity and a broad downregulation of apical transporters (including sodium, glucose, amino acid, and urate uptake). Furthermore, in pluripotent stem cell-derived cardiomyocytes, p-cresol sulfate incubation reduced contractility and increased the expression of growth/differentiation factor 15 (GDF15), a heart-derived hormone recently proposed as cardiovascular biomarker [24, 25], linked to subclinical atherosclerosis and higher mortality risk in hemodialysis (HD) population [26, 27].

**Figure 1:**
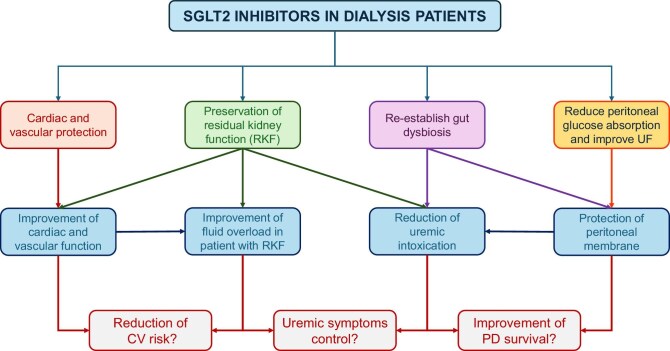
Potential benefits of SGLT2 inhibitors in patients undergoing HD or PD.

These pleiotropic effects of SGLT2i, together with other proposed off-target mechanisms [28], represent the rationale for supporting their use in patients with advanced CKD to prevent cardiovascular morbidity and mortality despite the tubular effect of SGLT2i in these patients is expected to be trivial. Furthermore, in patients receiving HD or peritoneal dialysis (PD), additional favorable effects of these drugs on residual kidney function (RKF) and peritoneal membrane (see next) can be hypothesized (Fig. 1).

## SGLT2i IN PATIENTS ON HEMODIALYSIS

Survival rate in both HD and PD patients remains low with the 5-year survival being ∼40% [29]. Cardiovascular disease is the main cause of death accounting for ∼50% of fatal events. Although cardiovascular deaths have declined in the general population, this trend is not seen in dialysis patients, particularly those <50 years, whose life expectancy is estimated to be >25 years shorter than the general population [30]. Unfortunately, clinical evidence on the use of SGLT2i so far available in patients undergoing HD has been limited to five studies with only 866 patients receiving SGLT2i (Table 1).

**Table 1:** Clinical effects of SGLT2i in ESKD patients.

Author, year [Ref]	Type of dialysis	Study design	Interventions	Patients (*N*)	Control group	Patients (*N*)	Follow-up	Outcome measures	Main results
Barreto J, 2023 [31]	HD and PD	Prospective, single-center	Dapagliflozin	7	Age/sex-matched controls with eGFR ≥60	7	7 days	Peak concentration of drug	No significant difference in peak concentration and AUC vs controls
Lin DS, 2025 [32]	HD	Single-arm, exploratory safety study	Empagliflozin	17	NO		4 weeks	Safety	Empagliflozin up to 25 mg/day is well tolerated
De La Flor JC, 2023 [33]	Incremental HD	Retrospective	Dapagliflozin or Empagliflozin	7	NO		12 months	KrU	Improvement in KrU
Wang CA, 2024 [37]	HD	Retrospective	SGLT2i	771	PS-matched	771	2 years	All-cause mortality	Significant reduced risk of mortality and MACE by 51% and 48%, respectively
Heerspink HJL, 2021 [38]	Not specified	Exploratory analysis of RCT	Dapagliflozin	66	Placebo	99	Not specified	All-cause mortality	Lower mortality with dapagliflozin after start of dialysis
Moral Berrio E, 2024 [41]	PD	Retrospective	Dapagliflozin or Empagliflozin	16	NO		6 months	Preservation of RKF	KrU and urinary output did not change
Lai JW, 2021 [42]	PD	Retrospective	Dapagliflozin or Empagliflozin	50	Not matched control	50	31 months	UF rate	Higher UF rate
Hamdan Z, 2024 [43]	PD	Retrospective	Dapagliflozin	20	NO		1 month	Peritoneal Equilibrium Test	No change in glucose peritoneal, sodium dip, UF, body weight
Doi Y, 2025 [44]	PD	RCT-crossover	Empagliflozin	36	Placebo	34	8 weeks	Change in UFV from baseline	UFV did not differ between treatments

AUC, area under the concentration-time curve; PS, propensity score; UF, ultrafiltration; UFV, ultrafiltration volume.

Evaluating SGLT2i efficacy and safety in HD first requires assessment of their pharmacokinetic profiles to exclude accumulation or excessive removal of drugs. In this regard, an interesting prospective, single-center, open-label trial assessed the dapagliflozin pharmacokinetic properties in patients treated from at least 3 months with regular HD (*n* = 5) or automated PD (*n* = 2) compared with seven age- and sex-matched controls with type 2 diabetes (T2DM) and preserved kidney function (eGFR ≥60 ml/min/1.73 m^2^) [31]. Median residual diuresis was 300 ml/day (IQR, 0–500). Pharmacokinetic profiles were evaluated after a single dose of dapagliflozin 10 mg (day 1) and after six daily doses of dapagliflozin to achieve steady state (day 7). The results confirmed that dapagliflozin is not dialyzable and showed no clinically significant accumulation (at steady state accumulation index was 26.7% in dialysis patients and 9.6% in controls). Pharmacokinetic profiles were similar for dapagliflozin and its inactive metabolite. These findings support a potential use of SGLT2i in this population since dialysis has no effects on pharmacokinetic properties of these drugs. Despite dapagliflozin was well tolerated by study participants, no firm conclusions on its safety in dialysis population can be drawn due to the small sample size and the short follow-up of the study.

A single-arm, open-label, dose-escalation study evaluated the effects of empagliflozin 5, 10, or 25 mg daily for 4 weeks in 17 patients on maintenance HD with heart failure (reduced or preserved ejection fraction). Due to its exploratory design, no pre-specified primary or secondary endpoints were defined [32]. This study reports first-time evidence of a beneficial electrophysiological effect of a short-term treatment with SGLT2i in patients under regular HD. Indeed, empagliflozin treatment was associated with significant shortening of QRS duration and stable QT intervals, and increase in serum calcium, without affecting blood pH or ketone levels. No safety concerns emerged, and the drug was well tolerated up to 25 mg/day without clinical adverse events.

An additional retrospective study evaluated SGLT2i efficacy in preserving RKF in seven diabetic patients who underwent incremental HD (1–2 sessions/week) during 12 months of follow-up [33]. In these patients, the preservation of RKF is mandatory to ensure the feasibility of incremental protocol and it is associated *per se* with better patient survival and health-related quality of life [34–36]. RKF, assessed as residual kidney urea clearance (KrU), increased with SGLT2i treatment from 4.91 ± 1.14 ml/min at baseline to 7.28 ± 1.68 ml/min at month 12 (*P *= .028) [33]. An improvement in volemic status of patients was testified by a decline of extracellular water/total body water ratio and a significant BP decline (from 148 ± 14/78±5 mmHg to 133 ± 12/70±7 mmHg at month 12) despite a reduction in antihypertensive treatment. The effective preservation of RKF was coupled with a good safety profile: only two episodes of urinary tract infections (UTI) were reported not requiring SGLT2i withdrawal. The small sample size and the lack of control group strongly limit the reliability of results (higher risk of bias) and their generalizability.

The largest study evaluating the association between SGLT2i and adverse outcomes is a retrospective cohort study of electronic health records in patients with T2DM at dialysis start [37]. Among 49 762 patients who initiated dialysis, 771 SGLT2i users within 3 months after dialysis were matched by propensity score with 771 non-users. Over a median follow-up of 2.0 years (IQR 0.3–3.9), SGLT2i users had a lower adjusted risk of MACE [hazard ratio (HR) 0.52, 95%CI 0.36–0.75, *P *< .001] and all-cause mortality (HR 0.49, 95%CI 0.34–0.69, *P *< .001). No significant differences were observed in the incidence of ketoacidosis, UTI or genital infection, hypoglycemia, dehydration, bone fractures, or amputations. These findings suggested the potential long-term cardiovascular protection and safety of SGLT2i use in T2DM patients receiving HD.

Finally, an exploratory analysis from the Dapagliflozin and Prevention of Adverse Outcomes in Chronic Kidney Disease (DAPA-CKD) trial reported that patients continuing dapagliflozin after dialysis start had a lower mortality rate in comparison with placebo-treated patients (17.6 and 25.3/100 patient-years, respectively) [38]. These results should be interpreted with caution given the *post hoc* design and the low number of patients who initiated dialysis (Table 1).

Overall, available evidence is weak mainly because studies are small, retrospective, or exploratory, with limited follow-up and heterogeneous endpoints. This limits the ability to draw firm conclusions about efficacy and safety of SGLT2i in HD population.

## SGLT2i IN PATIENTS ON PERITONEAL DIALYSIS

The main goals of optimal PD are to preserve RKF, reduce cardiovascular risk, and protect the peritoneal membrane [39, 40]. The successful achievement of these goals with SGLT2i remains poorly investigated with four clinical studies including only 124 PD patients (Table 1).

A retrospective, single-center study evaluated the potential effects of SGLT2i on the preservation of RKF in patients undergoing PD [41]. The basal mean RKF (1.5 ± 0.7 l/day) and KrU (4.1 ± 2.5 ml/min) remained unchanged during the 6 months of treatment with SGLT2i in both diabetic and non-diabetic patients. Notably, systolic BP significantly decreased from 140 ± 10 to 130±7 mmHg. Only two patients experienced adverse events (hypoglycemia and UTI). In a further retrospective study, 11 patients with T2DM continuing SGLT2i after starting PD were compared with 39 incident patients that during the same period discontinued SGLT2i [42]. During 31 months of follow-up, patients receiving SGLT2i had higher ultrafiltration (1322±200 vs. 985±415 ml/day, *P *= .013) without differences in BP, overall survival, technical survival, and UTI incidence. Authors described higher hemoglobin levels (11.2 ± 1.7 vs. 10.2 ± 1.7 g/dl) but data on anemia treatment were not reported. In a third retrospective study of 20 patients on continuous ambulatory PD, 1 month of dapagliflozin therapy did not significantly reduce peritoneal membrane glucose absorption or affect ultrafiltration [43]. The retrospective designs of these three studies are prone to bias due to the presence of confounding, and are poorly generalizable.

The only trial in PD population is a small multicenter, randomized crossover study from Japan, including 37 patients with chronic heart failure (HF) in PD for >3 months treated with empagliflozin 10 mg/day or placebo with random sequence separated by a 4-week washout period [44]. The primary outcome was the change in daily ultrafiltration from baseline to week 8. The authors found that empagliflozin as compared with placebo did not change the ultrafiltration volume with a between-treatment difference at week 8 of −38 ml/day (95% CI, −120 to +44; *P *= .36). This result was consistent across several pre-specified categories. A slightly but not significant higher incidence of adverse events was reported during empagliflozin treatment.

Beyond the cardiac benefits of SGLT2i that could theoretically support the use of these drugs in ESKD, in the subgroup of patients undergoing PD an additive effect on the peritoneal membrane should be considered based on experimental data. Indeed, SGLT2i could exert a potential role in modulating glucose reabsorption through the peritoneum from glucose-based dialysate, helping to maintain the osmotic gradient of glucose-containing PD solutions overtime and to improve ultrafiltration. Additionally, reducing glucose absorption by SGLT2i could also enhance carbohydrate metabolism in patients with T2DM, thereby improving glycemic control and preventing weight gain. Therefore, SGLT2i could provide both systemic and local benefits by inhibiting glucose reabsorption through the peritoneal membrane [39].

**Figure 2:**
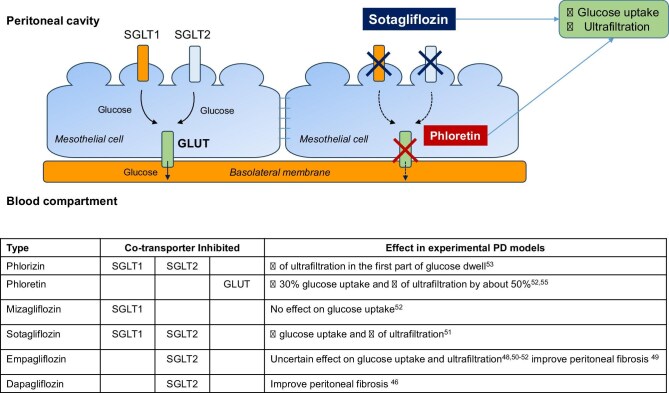
Hypothetical distribution on peritoneal membrane and potential mechanism of action of drugs acting on glucose transporters based on the experimental studies.

The potential effect of SGLT2i on the peritoneal function stems from the hypothesis that SGLT2 transporters are present on the peritoneal mesothelial cells. However, their localization remains unclear and experimental studies in animals and human cells provided contrasting findings. One study reported SGLT1 and Glucose Transporter Type 1 and Glucose Transporter Type 3 mRNA expression in human peritoneal mesothelial cells [45], while a more recent study disclosed the presence of SGLT2 protein in human peritoneal biopsies [46]. It has also been reported that SGLT2 expression is upregulated in response to longer PD duration and peritoneal exposure to glucose dialysate [47, 48], suggesting that increased presence of SGLT2 components on mesothelial cells might be a regulatory response to environmental stimuli. Similarly, data on the effect of SGLT2i on glucose peritoneal transport provided inconsistent results. Some experimental studies suggest that intraperitoneal exposure to selective SGLT2i (empagliflozin) may affect glucose uptake and ultrafiltration [49] and may ameliorate peritoneal fibrosis by suppressing TGF-β/Smad signaling and improving vascular remodeling [47, 50]. Conversely, a study in a PD model in uremic rodents reported reduced glucose uptake only with non-selective SGLT1/2 inhibitors (sotagliflozin) while no effect was measured with empagliflozin [51]. Similarly, no effect had been previously reported in animals treated with selective SGLT2i (empagliflozin [52]), nor with mizagliflozin, which is a selective SGLT1i [53], whereas an amelioration of ultrafiltration was reported with phloretin, a GLUT inhibitor [53, 54], suggesting that the beneficial effect is linked to the inhibition of GLUTs rather than SGLT2 transporters (Fig. 2) [55]. Data in humans align with this hypothesis [44]. Low-dose intraperitoneal phloretin administration in experimental PD model in rats significantly increased ultrafiltration suggesting a potential therapeutic use for patients with ultrafiltration insufficiency [56]. Overall, experimental evidences may support the usefulness of SGLT2i for the function of the peritoneal membrane but this hypothesis remains speculative given the inconsistent and limited clinical data available.

### Future perspectives

Several studies on SGLT2i efficacy in the dialysis population are forthcoming (Table 2). Most are short-term RCTs (8 weeks up to 12 months) or observational studies, which cannot establish causality, and primarily assess intermediate outcomes, mainly cardiac markers and echocardiographic parameters, recognized as risk factors for cardiovascular and all-cause mortality in ESKD patients. The most intriguing and promising study is certainly the Renal Lifecycle trial [57]. This pragmatic, multicenter, investigator-initiated, randomized, placebo-controlled clinical trial will enroll ∼1500 patients with ESKD (including HD, PD, eGFR ≤25 ml/min/1.73 m^2^ or patients with a kidney transplant and an eGFR ≤45 ml/min/1.73 m^2^). These patients will be randomized 1:1 to receive either dapagliflozin 10 mg per day or matching placebo to disclose difference on a composite outcome of kidney failure, hospitalization for HF or all-cause mortality over a follow-up of 4 years.

**Table 2:** Ongoing studies on the effect of SGLT2i in dialysis population.

Trial number	Intervention (acronym)	Country	Study design	Estimated number	Study duration	Target population	Primary outcome	Estimated completion
Mortality								
NCT05374291	Dapagliflozin 10 mg (Renal Lifecycle)	Europe, Singapore, Australia	RCT	1500	48 months	eGFR ≤25 ml/min/1.73 m^2^, HD or PD for ≥3 months, transplant with eGFR ≤45 ml/min/1.73 m^2^	Composite of all-cause mortality, kidney failure, and hospitalization for HF	Jan 2027
Feasibility								
NCT05614115	Empagliflozin 10 mg or 25 mg TIW	USA	RCT	75	12 weeks	HD treatment for ≥3 months	Persistence in follow-up, adherence to the full dose	Jun 2025
NCT05687058	Empagliflozin 10/day or 25 mg/TIW	USA	OS, two arms	24	3 months	HD or PD	Feasibility	Dec 2025
NCT05141552	Dapagliflozin 10 mg	China	RCT	20	12 weeks	HD for ≥3 months	Hypoglycemia or UTI	Jul 2022
NCT05309785	SIP-AkiD-1: Canagliflozin 300 mg SIP-AkiD-2: 100 mg	Canada	OS, single arm	44	26 weeks (SIP-AkiD-1) 8 days (SIP-AkiD-2)	eGFR <30 ml/min/1.73 m^2^ and ACR >200 mg/g (SIP-AkiD-1) or HD for ≥3 months (SIP-AkiD-2)	Change of ACR (SIP-AkiD-1) Pharmacokinetics (SIP-AkiD-2)	Jun 2026
Kidney outcomes								
NCT05196347	Dapagliflozin 10 mg (DAPA-advKD)	China	Open-label RCT	180	96 weeks	eGFR: 10 to 30 mL/min/1.73 m^2^	eGFR decline	Aug 2024
NCT05715814	Empagliflozin 25 mg (CANARY)	Canada	OS, single arm	20	2 weeks	PD with RKF (≥250 cc/day)	Change in measured GFR	Dec 2025
Cardiac outcomes								
ChiCTR2300073169	Henagliflozin 5 mg (HELD-HF)	China	RCT	112	24 weeks	HD or PD for ≥3 months with diagnosis of HFrEF	Change in LVMI measured by echocardiography	Feb 2024
NCT06182839	Canagliflozin 300 mg (CARe-MRI)	Canada	RCT	92	12 months	eGFR <20 ml/min/1.73 m^2^, HD or PD AND LVH or hospitalization for HF or ASCVD in the last 12 months	Left ventricular mass to volume ratio (cardiac MRI)	Mar 2029
NCT05737186	Dapagliflozin 10 mg	China	RCT	40	12 weeks	eGFR <20 ml/min/1.73 m^2^, HD or PD AND HFrEF	Quality of life in patients with chronic (HFrEF) and severe CKD	Apr 2024
NCT05179668	Dapagliflozin 10 mg (DAPA-HD)	Austria	RCT	108	6 months	HD for ≥3 months	Changes of LVMI (cardiac MRI)	Sep 2025
NCT06929169	SGLT2 inhibitor (SGLT2-HD)	Argentina	RCT	80	12 months	Online HDF for ≥3 months	Change in LVMI and LVEF (cardiac MRI and/or echocardiography)	Aug 2026
NCT06249932	Empagliflozin 25 mg (EMPA-RRED)	Taiwan	RCT	95	24 weeks	Dialysis with diagnosis of HFrEF	Left ventricular mass (cardiac MRI)	Dec 2030
NCT05685394	Dapagliflozin 10 mg (DARE-ESKD-2)	Brazil	RCT	80	6 months	HD or PD for ≥3 months	NT-proBNP	Dec 2024
NCT05967156	Empagliflozin 10 mg	Iran	OS, single arm	30	1 month	HD with RKF and HF	BNP	Mar 2025
NCT05786443	Empagliflozin 10 mg (SEED)	USA	RCT	60	12 weeks	Maintenance HD with RKF	Changes in ECV, ICV, TBW, 24 h urine volume	Dec 2026
NCT06249945	Empagliflozin 25 mg (EMPA-PRED)	Taiwan	RCT	150	24 weeks	Dialysis with diagnosis of HFpEF	E/A ratio	Dec 2030
jRCT1031230624	Empagliflozin 10 mg	Japan	Open-label RCT	40	24 weeks	PD for ≥3 months	Change in ECW measured by BIA	Dec 2027
Peritoneal outcomes								
NCT05671991	Empagliflozin 10 mg or 25 mg (EMPA-PD)	USA	RCT-crossover	30	8 weeks	PD for ≥3 months	Peritoneal glucose absorption	Jun 2026
NCT06913647	Canagliflozin 300 mg (CAN-PD)	Canada	RCT-crossover	30	10 weeks	PD for ≥3 months	Change in the ratio of intraperitoneal glucose	Sep 2028
NCT05250752	Dapagliflozin 10 mg (PRESERVE)	Denmark	OS, single arm	10	21 days	PD for ≥14 days	Peritoneal glucose uptake	Dec 2022

OS, observational study; HFpEF, heart failure with preserved ejection fraction; HFrEF, heart failure with reduced ejection fraction; E/A ratio: Mitral early (E) and late (A) diastolic filling velocity ratio; MRI, magnetic resonance imaging; LVH, left ventricular hypertrophy; ASCVD, atherosclerotic cardiovascular disease; BNP, brain natriuretic peptide; ECV, extracellular volume; ICV, intracellular volume; TBW, total body water; ACR albumin creatinine ratio; HDF, hemodiafiltration; LVMI, left ventricular mass index; LVEF, left ventricular ejection fraction.

A Canadian single-arm study (the SIP-AkiD-1) will assess the safety and renal outcomes (GFR decline and albuminuria) in 44 patients with advanced CKD before (<30 ml/min/1.73 m^2^ and ACR >200 mg/g) treated with 300 mg of canagliflozin. A sub-study (the SIP-AkiD-2) will be performed in patients on maintenance HD for at least 3 months to evaluate pharmacokinetic of a short treatment with canagliflozin 100 mg.

Renal outcomes will be specifically investigated by a phase-3 trial (DAPA-advKD trial) performed on patients with advanced CKD (eGFR 10–30 ml/min/1.73 m^2^) to evaluate the effect of dapaglifozin 10 mg in comparison with standard care on eGFR decline over 2 years. Furthermore, a single-arm study will evaluate changes in RKF in patients with PD and residual GFR >250 ml/day treated with empagliflozin 25 mg for 2 weeks.

Cardiac outcomes will be focused on several studies exploring the effect of SGLT2i in patients with advanced CKD. Interesting data will be provided in the near future by the dapagliflozin cardiovascular effects on end-stage kidney disease (DARE-ESKD-2) trial (NCT05685394) that was completed on December 2024 [58]. The primary aim of this RCT is to investigate the myocardial effects of dapagliflozin 10 mg versus placebo for 6 months in patients undergoing dialysis by evaluating changes of NT-proBNP. Furthermore, this study will provide a comprehensive evaluation on the potential role of dapagliflozin also in echocardiographic patterns, physical performance (6-min walking test), HF symptoms (Kansas City Cardiomyopathy Questionnaire, KCCQ), and body composition (dual X-ray absorptiometry, bioimpedance).

Four RCTs (CARe-MRI, DAPA-HD, SGLT2-HD, EMPA-RRED) will assess the effect of either dapagliflozin, empagliflozin, or canagliflozin on left ventricular mass measured by either echocardiography of MRI (Table 2). In addition, a multicenter, randomized, double-blind, placebo-controlled trial has evaluated the effect of henagliflozin 5 mg (an SGLT2i approved in China) on echocardiography-measured left ventricular mass index in 112 dialysis patients (54% in HD and 46% in PD) with HF with preserved ejection fraction [59]. Results recently reported in abstract form showed a significant reduction of left ventricular mass index after 24 weeks of henagliflozin without changes in atrial volume, E/e’ and NT-proBNP [60].

## CONCLUSIONS

In the absence of robust data on the efficacy and safety of SGLT2i in patients with advanced CKD and ESKD it is wise at present to restrict their use in these populations. However, experimental data and preliminary clinical observations emphasize the possible protective role of this class of drugs in very risky phases of disease history, as the transition phase from CKD to ESKD [61, 62] and the first 90 days from dialysis start [63]. Future trials will fill the gap in the knowledge on the ability of SGLT2i to improve cardiopulmonary conditions and long-term prognosis in those patient categories that, despite carrying the highest cardiovascular and mortality risk, actually cannot benefit from this drug class.

## Data Availability

There are no new data associated with this article.
